# A Methylation-Based Reclassification of Bladder Cancer Based on Immune Cell Genes

**DOI:** 10.3390/cancers12103054

**Published:** 2020-10-20

**Authors:** Qizhan Luo, Thomas-Alexander Vögeli

**Affiliations:** Department of Urology, University Hospital RWTH Aachen, RWTH Aachen University, Pauwelsstrasse 30, 52074 Aachen, Germany; qizhan.luo@rwth-aachen.de or

**Keywords:** immune cell infiltration, DNA CpGs, bladder cancer, classification, subtype, subgroup, mutation, copy number variation (CNV), tumor microenvironment, immune checkpoints, immunotherapy and chemotherapy, targeted drug therapy, stem cell, inflammation, endothelial cells, fibroblasts, PD-L1

## Abstract

**Simple Summary:**

Bladder cancer (BC) development is highly related to immune cell infiltration. In this study, we aimed to construct a new classification of bladder cancer molecular subtypes based on immune-cell-associated CpG(Methylation) sites. The classification was accurate and stable. BC patients were successfully divided into three subtypes based on the immune-cell-associated CpG sites. The clinicopathologic features, distribution of immune cells, level of expression of checkpoints, stromal score, immune score, ESTIMATEScore, tumor purity, APC co_inhibition, APC co_stimulation, HLA, MHC class_I, Type I IFN_respons, Type II IFN response, and DNA stemness score (DNAss) presented significant differences among the three subgroups. The specific genomic alteration was also different across subgroups. High-level immune infiltration showed a correlation with high-level methylation. A lower RNA stemness score (RNAss) was associated with higher immune infiltration. Cluster 2 demonstrated a better response to chemotherapy. The anti-cancer targeted drug therapy results are different among the three subgroups.

**Abstract:**

Background: Bladder cancer is highly related to immune cell infiltration. This study aimed to develop a new classification of BC molecular subtypes based on immune-cell-associated CpG sites. Methods: The genes of 28 types of immune cells were obtained from previous studies. Then, methylation sites corresponding to immune-cell-associated genes were acquired. Differentially methylated sites (DMSs) were identified between normal samples and bladder cancer samples. Unsupervised clustering analysis of differentially methylated sites was performed to divide the sites into several subtypes. Then, the potential mechanism of different subtypes was explored. Results: Bladder cancer patients were divided into three groups. The cluster 3 subtype had the best prognosis. Cluster 1 had the poorest prognosis. The distribution of immune cells, level of expression of checkpoints, stromal score, immune score, ESTIMATEScore, tumor purity, APC co_inhibition, APC co_stimulation, HLA, MHC class_I, Type I IFN Response, Type II IFN Response, and DNAss presented significant differences among the three subgroups. The distribution of genomic alterations was also different. Conclusions: The proposed classification was accurate and stable. BC patients could be divided into three subtypes based on the immune-cell-associated CpG sites. Specific biological signaling pathways, immune mechanisms, and genomic alterations were varied among the three subgroups. High-level immune infiltration was correlated with high-level methylation. The lower RNAss was associated with higher immune infiltration. The study of the intratumoral immune microenvironment may provide a new perspective for BC therapy.

## 1. Introduction

Recently, diverse immunotherapy methods have been proven to successfully treat numerous lethal cancers [1]. These included cytokine treatment, cellular therapy, immune checkpoint blockades, and therapeutic vaccines [2]. Immune checkpoint inhibitors showed remarkable anti-tumor functions in several human cancers, including programmed cell death protein 1 (PD-1), cytotoxic T lymphocyte antigen-4 (CTLA-4), and PD-1 ligand (PD-L1) antibodies [3,4,5]. The FDA has approved two cytokines as anti-tumor agents against kidney cancer and metastatic melanoma [6]. Preventive and therapeutic anticancer vaccines have a significant anti-tumor function in several cancers, such as hepatitis B virus vaccine [7], Sipuleucel-T, human papillomavirus vaccine [8], and GVAX vaccine [9,10]. There is a remarkable heterogeneity in the response rates to treatment across individuals, yet not all immunotherapy is successful in treating patients [2].

A previous study also revealed the interaction of various immune cells and signaling pathways between the tumor and immune cells [11]. There are several types of immunotherapy strategies to treat bladder cancer (BC); for instance, treating high-risk non-muscle invasive bladder cancer (NMIBC) with intravesical administration of the Bacillus Calmette-Guerin (BCG) [12]. BCG is a standard practical therapy in NMIBC [13], but, unfortunately, 25–45% of patients with high-risk papillary tumors or carcinoma in situ did not benefit from BCG therapy [14]. Several immune checkpoint inhibitors have been utilized to treat BC. Among them, atezolizumab, avelumab, durvalumab, nivolumabis, and pembrolizumab were recommended for patients with advanced or metastatic tumors [15,16]. In addition, the inhibition of CTLA-4, including ipilimumab and tremelimumab, was suggested to increase the immune response of BC [17]. Finally, intravesical interleukin 12 (IL-12) activates the immune system and weakens the status of immunosuppression in tumor cells [18]. However, not all patients have the same response to the above kinds of therapy [17]. Similarly, research reported that only approximately 20% of patients can benefit significantly from immunotherapy [19,20]. Therefore, accurate classification methods must be developed to help to enhance the optimal scheme of BC patients’ responses to immunotherapy.

Tumor infiltrating lymphocytes (TIL) have an important role in the chemotherapy response and in enhancing the clinical effect in all subtypes of breast cancer [21]. Furthermore, two previous studies proved that high immune cell infiltration was associated with a favorable prognosis after chemotherapy [22,23]. Besides, two previous studies reported that the basal subtype of muscle invasive bladder cancer with immune infiltration had a sensitivity to chemotherapy [24,25].

The treatment response and prognosis of patients were predicted by immune cells with the current molecular stratification of BC patients [26]. In contrast to that study, our study had some differences as follows. The first difference is that we analyzed based on multi-omics, including DNA methylation, RNA, DNA mutations, and copy number variations. The second difference is that we divided the bladder cancers into three subtypes based on methylation. The third difference is that they did not show any clinical implications regarding their classification. In our study, BC was divided into three distinct subtypes based on immune cell-related methylation site profiles. The three methylation site subtypes are associated with different molecular features, cellular properties, and clinical outcomes. In fact, the classification of immune-related methylation site subtypes may help to enhance the optimal scheme of BC patients that are responsive to immunotherapy.

## 2. Results

### 2.1. Three Subgroups Based on Differentially Methylated Sites (DMSs)

**Figure 1:**
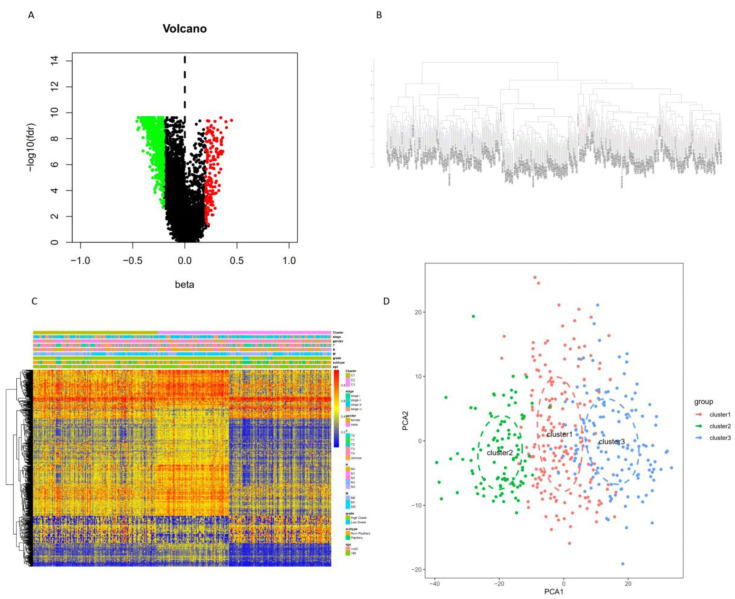
Three subgroups based on differentially methylated sites. (**A**). 715 DMSs between normal samples and bladder cancer samples. (**B**). The consensus clustering of 715 DMSs was divided into three CpG subgroups. (**C**). The heatmap of methylation based on 715 DMSs. (**D**). Principal component analysis (PCA) validated the stability of the classification.

Seven hundred and eighty-two immune cell biomarker-associated genes were selected from previous studies [27], and 8703 corresponding immune cell biomarker-associated methylation sites were acquired. The parameter of infiltration was an adjusted *p*-value < 0.05 and |deltabeta| > 0.2. Seven hundred and fifteen DMSs were identified between normal samples and tumor samples (Figure 1A). Probes revealing the *p*-value < 0.05 and deltabeta > 0.2 were defined as hypermethylated, and those with a *p*-value < 0.05 and deltabeta < 0.2 were defined as hypomethylated. A total of 553 DNA hypomethylation sites and 162 hypermethylation sites were obtained.

### 2.2. Classification of Methylation Subtypes of BC

The consensus clustering of 715 DMSs was classified into three subtypes (Figure 1C). Cluster 1 showed mid-range-methylation, cluster 2 showed the highest methylation level, whereas cluster 3 had the lowest methylation. Principal component analysis (PCA) was utilized to check the stability of the consensus classification (Figure 1D).

**Figure 2:**
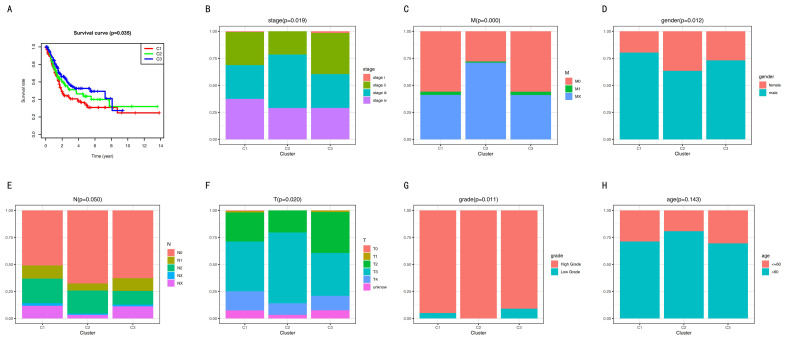
The overall survival (OS) curve and clinicopathologic features. (**A**) Overall survival curve. (**B**–**H**) Clinicopathologic features among the three subgroups.

The overall survival (OS) curve of BC subsets was obtained using the Kaplan–Meier method (Figure 2A). Cluster 1 had the poorest prognosis. Cluster 3 had the best prognosis. Next, we carried outonducted a log-rank test between each pair of subtypes and found a significant difference only between clusters 1 and 3 (*p*-value was 0.009). However, several studies reported that it was unnecessary that there was a significant difference between each pair of clusters [25,28,29,30]. Furthermore, the sub-sequence analysis showed that the bio-mechanism had significance, and the clinical significance was different among the three subgroups. Therefore, we divided the BC into three clusters.

In our study, a barplot demonstrates the relationship between the clinical traits and the biological characteristics of the subtypes (Figure 2B–H). Excluding age, the other clinicopathologic features had significant differences among the three subgroups. Our results show that Cluster 3 had more stage I, more low-grade types, and less T3.

### 2.3. Identifying Different Methylation Levels and Distinct Gene Expression Levels of the Different Subgroups

**Figure 3:**
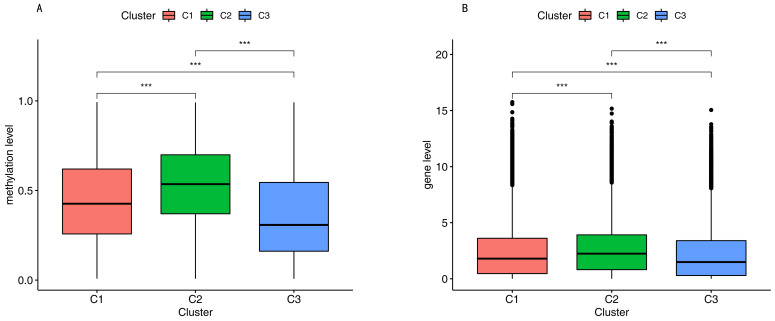
Methylation level and gene level. (**A**). Gene level among the three subgroups. (**B**). Methylation level among the three subgroups. Three asterisks indicate a *p*-value less than 0.001.

We compared the DNA methylation level and immune gene levels among the three subgroups using the chi-square test with *p*-value < 0.05. Figure 3A shows the differentially immune cell biomarker-associated methylation levels. Cluster 1 revealed a mid-range-methylation level, cluster 2 revealed the highest methylation level, and cluster 3 revealed the lowest methylation level. The results in Figure 3A are consistent with Figure 1C. The DNA methylation levels provided significant differences among the three subgroups. We also found significantly different immune gene levels across the three subgroups (Figure 3B).

The methylation expression in one subgroup was compared with the rest of the subgroups using the Wilcox test. Consequently, we found 14, 540, and 136 methylation sites with higher expression levels in cluster 1, cluster 2, and cluster 3, respectively. Fourteen methylation sites overlapped between subtypes cluster 1 and cluster 2. However, no overlapped methylation sites were identified in the remaining cluster-pairs.

### 2.4. Immune in Different Subgroups

**Figure 4:**
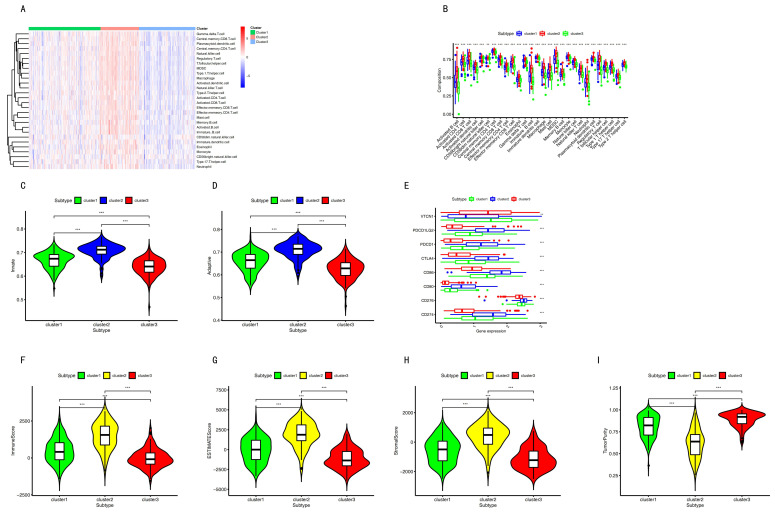
Immune status among the three subgroups. (**A**) Immune cell infiltration among the three subtypes. (**B**). Immune cells the among three subgroups. (**C**,**D**) Innate immune cells and adaptive immune cells among the three subgroups. (**E**) Immune checkpoints among the three subsets. (**F**–**I**) The immune microenvironment among the three groups. Three asterisks indicate a *p*-value less than 0.001. Two asterisks indicate a *p*-value less than 0.01.

In Figure 4A, cluster 1 had mid-range immune infiltration. The correlation with high immune infiltration is shown in cluster 2, unlike cluster 3, which had low immune infiltration. The immune infiltration was compared among the three subtypes, and there were remarkable differences among these subtypes (Figure 4B). Evidently, the immune checkpoints demonstrated significant differences among these subtypes (Figure 4E).

### 2.5. Tumor Microenvironment (TME)

The tumor microenvironment contains stromal cells, tumor cells, and immune cells. The higher the stromal score and immune score, the lower the purity of the tumor. As shown in Figure 4F–I, cluster 2 presented the highest stromal score, immune score, ESTIMATEScore, and the lowest purity of tumor. On the contrary, cluster 3 had the lowest stromal score, immune score, ESTIMATEScore, and the highest tumor purity.

### 2.6. Single Sample Gene Set Enrichment Analysis (ssGSEA)

**Figure 5:**
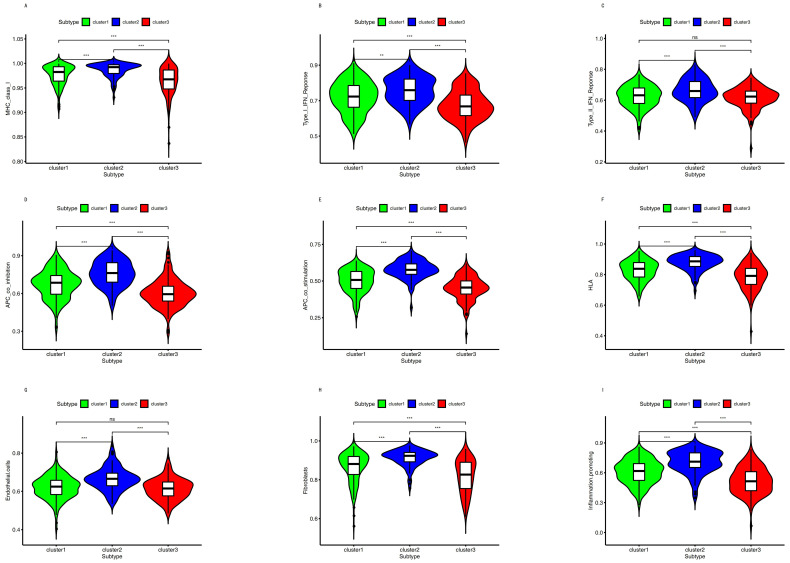
Immune-related molecular biomarkers among the three clusters. (**A**) MHC_class_I. (**B**) Type_I_IFN_Response. (**C**) Type_II_IFN_Response. (**D**) APC_co_inhibition. (**E**) APC_co stimulation. (**F**) HLA. (**G**) Endothelial cells. (**H**) Fibroblasts. (**I**) Inflammation-promoting. Three asterisks indicate a *p*-value less than 0.001. Two asterisks indicate a *p*-value less than 0.01. Ns indicates no significance.

The biomarkers of APC co_inhibition, APC co_stimulation, endothelial cells, fibroblasts, HLA, inflammation-promoting, MHC class_I, Type I IFN_Response, and Type II IFN Response were significantly different among these subtypes (Figure 5).

### 2.7. Comparing with the Other Classification

**Figure 6:**
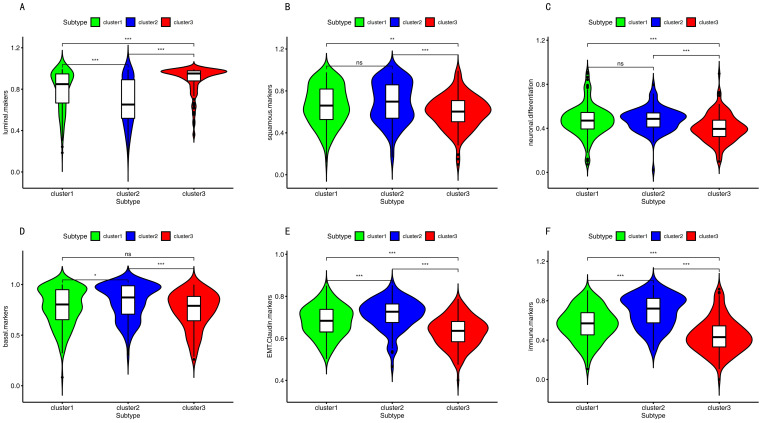
The bio markers of other subtypes among three subgroups. (**A**–**F**) The biomarkers of other subtypes among three subgroups. Three asterisks indicate a *p*-value less than 0.001. Two asterisks indicate a *p*-value less than 0.01. One asterisk indicates a *p*-value less than 0.05. Ns indicates no significance.

As shown in Figure 6, cluster 3 had the highest luminal marker expression and the lowest squamous and neuronal differentiation marker expression. Cluster 2 had the highest basal and EMT Claudin marker expression.

### 2.8. DNAss and RNAss among Subgroups

**Figure 7:**
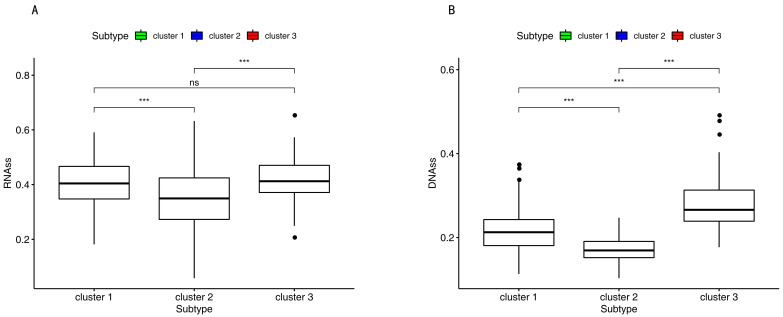
RNA stemness score and DNA stemness score. (**A**,**B**) RNAss and DNAss among the three subgroups. Three asterisks indicate a *p*-value less than 0.001. Ns indicates no significance.

The DNA hypermethylation of those promoter genes suppressed the gene expression, which, in turn, benefited the cancer cells. Therefore, down-regulation of those genes may lead to the occurrence of cancer stem and progenitor cells by DNA hypermethylation [31,32]. RNAss and DNAss were the lowest in cluster 2 in Figure 7.

### 2.9. Analysis of Mutations and CNVs among the Three Subgroups

**Figure 8:**
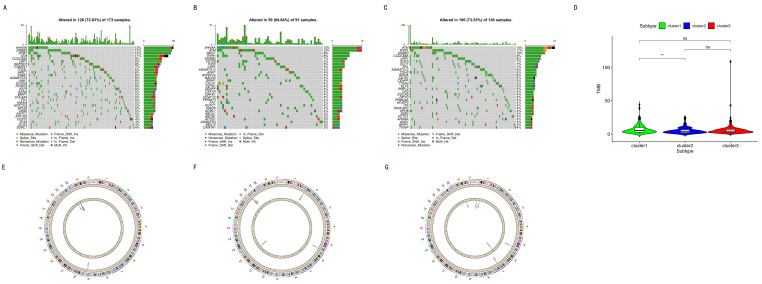
Mutations and CNV. (**A**) Immune-cell-associated gene mutations in cluster 1. (**B**) Immune-cell-associated gene mutations in cluster 2. (**C**) Immune-cell-associated gene mutations in cluster 3. (**D**) TMB among the three subtypes. Two asterisks indicate a *p*-value less than 0.01. Ns indicates no significance. (**E**–**G**) Immune-cell-associated gene CNVs in the three subgroups.

A total of thirty immune-cell-associated genes with the highest mutation proportion in each subtype are shown in Figure 8A–C. A further fifty-eight immune-cell-associated genes were identified from the above thirty genes in each subgroup. Together, the results show that there was less overlap among the three subtypes (Figure 8A–C). In additional, the mutations of ITGA9, ENG, EVI5, ATIC, and FZD2 in cluster 1 were significantly higher than those in other subtypes. The mutations of CTSZ, HOXA1, and KLRF1 in cluster 2 were significantly higher as well compared with those in other subtypes. Likewise, the mutations of DLC1, OSBPL1A, RRP12, C3AR1, MPZL1, and ITK in cluster 3 were significantly higher than those in other subtypes. TMB had a significant difference only between cluster 1 and cluster 2. (Figure 8D).

Finally, the CNV data were analyzed, and 391 normal tissue and 410 tumor tissue were extracted. In a comparison of CNV data of one group with the other two groups (cluster one, two, and three) (Figure 8E), the results were as follows. The CNV data in one subgroup were compared with the other two subgroups. One gene with significant copy number gains was in cluster 1, and three genes with significant copy number losses were in cluster 1. Figure 8F shows four genes with significant copy number gains and one gene with significant copy number losses in cluster 2. In Figure 8G, there are two genes with significant copy number gains and four genes with significant copy number losses in cluster 3.

### 2.10 Clinical Implications Regarding Our Classification

We analyzed chemotherapy’s impact on the three subgroups. Three hundred and ninety-eight cases were treated using chemotherapy in this study. Almost half of the unknown therapy information was in cluster 3 (Table 1), and cluster 3 had the highest luminal biomarker expression and lowest immune infiltration, which demonstrated that the luminal subtype had a good survival rate with and without neoadjuvant chemotherapy [33]. Therefore, we only compared the chemotherapy information and overall survival rate between cluster 1 and cluster 2. In this study, 80.2% of patients in cluster 2 received chemotherapy, and 95.9% of patients in cluster 1 received chemotherapy.

CPD represents clinical progressive disease, CR represents complete response, PR represents partial response, and SD represents stable disease. In patients of cluster 2, 59.7% reached a CR after chemotherapy, while in patients of cluster 1, only 32.9% reached a CR. We found that 62.3% of patients of cluster 2 had a response to chemotherapy, which included CR and PR; However, 53.2% of patients in cluster 1 had a response to chemotherapy. In addition, Kaplan–Meier analyses showed that cluster 2 had more improvement in the overall survival after chemotherapy (Table 2 and Figure 1). All the above results demonstrate that cluster 2 had a higher improvement in the overall survival after chemotherapy.

## 3. Discussion

In recent years, there has been increased interest in DNA methylation alteration as altered DNA methylation patterns are hallmarks of tumors. Typically, unmethylated promoters may change into densely methylated forms such as tumor suppressors which will facilitate gene silencing [32]. Other sequences may alter into hypomethylated forms in tumors, which results in the abnormal activation of genes that are usually suppressed by DNA methylation [34]. Hypermethylation events have also been reported to be biomarkers of human tumors, for an early examination of blood, urine, and other body fluids for prediction of the response and prognosis of treatment and for monitoring cancer recurrence [35].

To understand the mechanism of cancer, help guide therapy, and improve prognoses, it is vital to identify accurate subtypes. Several studies reported identifying subtypes based on DNA methylation, including colon adenocarcinoma [36], cervical cancer [37], glioblastoma [38], and bladder cancer [39]. This study divided BC into three distinct subtypes based on the immune-cell-related methylation profiles (Figure 1B–C). To check for the stability and probability of the classification, PCA was utilized to validate the stability of the classification (Figure 1D), thus proving that the classification was stable and accurate. The three immune subtypes were related to different clinical results (Figure 2). The methylation levels among the three subtypes were significantly different. The distribution of immune cells, genes corresponding to specific DNA methylation sites, level of expression of checkpoints, stromal score, immune score, ESTIMATEScore, APC_co_inhibition, APC_co_stimulation, HLA, MHC_class_I, Type_I_IFN_Response, and Type_II_IFN_Response had significant differences among the three subgroups. All were used to verify the stability and accuracy of the classification (Figure 4 and Figure 5).

A meta-analysis proved that the DNA methylation trended toward a poor clinicopathological result [40]. However, they found no correlation with age [40], which was the same in our study. An abnormal promoter methylation level was correlated with clinicopathological profiles in BC [41]. Researchers found that higher methylation values of four genes (RASSF1A, CDH1, CDH13, and APC) were significantly associated with several traits of poorer outcome (tumor stage, growth pattern, muscle invasion, and tumor grade). Clearly, our study was similar to the previously mentioned studies (Figure 2). Cluster 2 had the highest methylation and several poor clinicopathological parameters, including less stage I and II, less M0, less T1 and T2, and no low-grade.

In this current study, different subtypes had different survival rates. This may vary due to the following reasons: (1) abnormal DNA methylation may lead to a poor prognosis in cancer patients [42]. The progression and prognosis of cancer may be affected by the hyper-methylation of DNA [43]. (2) Tumor cells in the microenvironment can express high levels of immunosuppressive cytokines to forbid T cell proliferation and activity while facilitating tumor development and progression [44,45]. Tumor-expressing specific molecules can be sufficient to induce immunosuppression and facilitate immune evasion [46]. Subtle changes in the compositions of immune cells can have different influences on tumor progression [47]. Previous studies reported that a high density of macrophages in the microenvironment was correlated with a poor prognosis in bladder cancer patients [48]. (3) Patients with the luminal subtype presented well with and without neoadjuvant chemotherapy [30,33]. In our study, cluster 3 demonstrated the lowest methylation level, low immune cell infiltration, more luminal biomarkers, and less neuronal differentiation biomarkers, which might indicate why it had a good survival rate (Figure 1C, Figure 3A, Figure 4A and Figure 6).

However, mid-range-methylation and mid-range immune infiltration were found in cluster 1, which had the worst survival. The highest methylation and the highest immune infiltration were found in cluster 2, which had intermediate survival. We might find the following reasons.

(1) Up-regulation of the VTCN1 expression in bladder cancer led to poor survival [49,50]. B7x (VTCN1) was remarkably overexpressed in many human cancers, and it repressed the antitumor immune effect and regulated the escape from immunosurveillance [51]. A high-level expression of CD80 and CD86 may result in a high survival benefit of patients with nasopharyngeal carcinoma [52]. The absence or low-level expression of CD80 and CD86 in cancers could be one of the mechanisms in which cancers escape immunosurveillance [52]. The checkpoints illustrated in Figure 4E. demonstrated significant differences among the three subgroups. Among them, VTCN1 (B7-H4) had the higher expression in cluster 1. On the contrary, CD80 and CD86 had the lower expressions in cluster 1.
(2) The frequency of gene mutation and the CNV were different among the three groups. There was little overlap of mutant genes among the three subtypes, as shown in Figure 8A–C. These CNV genes among the three subgroups were completely different (Figure 8E).
(3) HLA plays an important role in the presentation of neoantigens [53,54]. Due to HLA loss, a tumor can escape immune monitoring [53,54].
(4) The downregulation of MHC class I expression also causes immune escape [55]. As shown in Figure 5, HLA and MHC class I had the highest expression in cluster 2.
(5) High immune infiltration improved the clinical outcomes from chemotherapy [23,56,57]. In this study, 80.2% of patients in cluster 2 received chemotherapy, and 95.9% of patients in cluster 1 received chemotherapy. Thus, all previously mentioned factors might cause a poorer overall survival rate in cluster 1 compared with cluster 2.

A previous study reported that muscle-invasive bladder cancer was divided into five subtypes based on the mRNA expression profiles [30]. The five subtypes were luminal-papillary, luminal-infiltrated, luminal, basal-squamous, and neuronal. The neuronal subtype had the poorest overall survival time [30]. However, the luminal-infiltrated subtype and basal-squamous subtype had medium-level overall survival times [30]. In the present study, cluster 3 had the highest luminal marker expression and the lowest basal and neuronal marker expression. Cluster 2 had the highest basal, immune, and EMT Claudin markers with more T-cell infiltration. The neuronal and squamous markers were the same in cluster 1 and cluster 2. Cluster 1 and cluster 2 with high neuronal marker expression demonstrated poorer survival times, as shown in Figure 6.

The relationship between methylation and immune infiltration is important. A previous study reported that the correlation between DNA methylation and gene expression in lung cancer was identified for approximately 750 genes [58]. They found that one third of these correlations were positive, which indicates the challenges in finding widespread and strong negative correlations between gene expression and genome-wide CpG methylation [58]. A previous study reported that the high methylation subgroup had low infiltration in skin cutaneous melanomas and breast cancer [59]. However, another study showed no distinct correlation between methylation and immune infiltration [60]. In addition, another study showed that one subgroup with low methylation had low infiltration [28]. Cong Liang et al. reported a high concordance between the methylation value and gene expression level, which predicted the immune infiltration levels in tumors [61]. All the above publications proved that the positive or negative correlation between immune infiltration and methylation level depended on the specific CpG sites. In the current study, cluster 2 with high methylation levels had a high immune infiltration (Figure 3).

The scores of stromal and immune cells were based on specific biomarkers associated with the infiltration of stromal and immune cells in the tumor samples. The stromal and immune scores formed ESTIMATE scores. These scores of stromal and immune cells were negatively correlated with the tumor purity. The ESTIMATE scores also had a negative correlation with the tumor purity [62,63]. In this study, cluster 2 had the highest immune infiltration and the highest stromal cells (Figure 4). Overall, cluster 2 had the highest stromal score, immune score, ESTIMATE scores, and the lowest tumor purity, in contrast to cluster 3 (Figure 4F–I). The distribution of immune scores among the three subgroups was consistent with the distribution of immune cells (Figure 4F–I).

Endothelial cells can remodel the local immune microenvironment and help tumor cells to escape immunosurveillance in different ways [64]. Endothelial cells not only release chemokines to promote leukocyte migration into tumor tissues, but also express adhesion proteins to facilitate peripheral leukocyte capture [65]. Thus, endothelial cells can forbid the activation and chemotaxis of immune cells and mediate inhibitory molecules to facilitate immune tolerance [66,67]. Endothelial cells also show an increased expression of PD-L1 to repress T cell activation [68,69,70]. In addition, FasL expression in endothelial cells promotes their ability to suppress the activation of CD8+ T cells, causing endothelial-cell-associated immune cell death and promoting tumor escape [71,72]. In the present study, the density of the endothelial cells in cluster 2 was the highest, and immune infiltration was the highest in cluster 2 (Figure 5). This implies that the endothelial cells might help tumor cells to escape immunity.

Cancer cells interacted with cancer-associated macrophages and tumor-associated fibroblasts, which promote tumor progression in bladder cancer [73]. In the present study, the distribution of fibroblasts among the three subgroups was consistent with the macrophage distribution (Figure 5). This indicates a correlation between fibroblasts and macrophages.

This can be an effective part of a patient’s cancer treatment regimen due to the numerous inflammatory molecules that play an important role in the progress and development of cancer [74]. The role of inflammatory molecules is far from being fully understood [74]. Chronic inflammation plays an important role in inducing aberrant methylation [75,76,77,78,79,80]. However, the molecular mechanisms are not yet understood [75,76,77,78,79,80]. A previous reviewer emphasized the importance of the inflammatory response in the recurrence risk and progression of BC [81]. The inflammatory cells and inflammatory cytokines in the chronic inflammatory microenvironment of solid tumors contributed to BC generation and progression via multiple mechanisms [81].

The proinflammatory cells, including immune cells, such as macrophages, myeloid-derived suppressor cells, regulatory T cells, dendritic cells, mast cells, neutrophils, and lymphocytes [81,82], belong to immune infiltration cells. In this study, the distribution of inflammation-promoting cells among the three subgroups was consistent with the distribution of immune cells (Figure 5). This demonstrates that the inflammation-promoting cells and immune cells might affect each other. However, the precise role of the inflammation promotion requires further study.

A study divided triple-negative breast cancer into three subgroups based on immune profiling [83]: the immunity-high group had the most HLA genes with higher expression, the immunity-mid had medium expression, and the immunity-low group had HLA genes with lower expression. Similarly, the immunity-high group also had high MIC class I, Type I IFN response, Type II IFN response, and APC [29,83,84]. However, the immunity-low group had MIC class I and APC with a lower expression [29,83,84]. In the present study, as shown in Figure 5, we found the same as the above studies.

In the present study, most checkpoints had the highest expression levels in cluster 2. There are several factors that affect the expression of checkpoints. First, aberrant methylation causes abnormal mRNA expression. The CD28, CTLA4, CD80, and CD86 expression values are mediated by DNA methylation [85]. The positive or negative correlation between the expression levels of those checkpoints and methylation levels depends on the specific CpG sites [85]. However, a previous publication showed a significantly positive correlation between the PD-L1 promoter methylation level and protein expression level in advanced gastric cancer [86]. Secondly, the distribution of immune checkpoints varied in different subtypes. A publication showed that the basal-squamous subtype had high CD274 (PD-L1) and CTLA4 expression [30]. Thirdly, high immune infiltration may be one of the factors. Several previous studies reported high immune infiltration with high LD-1(PDCD1) expression [29,83,84]. Similarly, another study reported that tumoral B7-H3 (CD276) overexpression was correlated with high tumor-infiltrating cytotoxic lymphocytes [87]. Two articles showed PDCD1, CD274, PDCD1LG2, CD86, CTLA4, and CD80 overexpression in the subtype with high immune infiltration [29,84].

The DNA hypermethylation of those promoter genes suppressed gene expression, which in turn gave the cancer cells the maximum benefits. Therefore, down-regulation of those genes may lead to the occurrence of cancer stem and progenitor cells by DNA hypermethylation [31,32]. The range of scores was from 0 to 1. Zero indicates high differentiation, and one indicates undifferentiation [88]. However, in this study, the RNA stemness score and DNA stemness score were the lowest in cluster 2 (Figure 7). This challenged the above findings in which down-regulation of those genes may lead to the occurrence of cancer stem and progenitor cells by DNA hypermethylation. The previous study found that for several tumor types, such as BLCA, LUSC, HNSC, and GBM, there was a negative correlation between DNAss and the leukocyte fraction and/or lower PD-L1 expression [88]. In this study, cluster 2 had the highest immune infiltration and a high-level expression of CD274 (Figure 4E); however, cluster 3 had the lowest DNAss. This result in the current study is the same as the previous work. In this study, we also found that the lower RNAss was associated with higher immune infiltration and a higher-level expression of CD274 (Figure 4E and Figure 7).

The high TMB, non-small-cell lung cancer group had more DNA methylation aberrations, including hypermethylation [89]. The correlation between TMB and the DNA methylation level was negative in head and neck squamous cell carcinomas [85]. Unlike the previous study, in the present study, TMB demonstrated a remarkable difference only between cluster 1 and cluster 2. (Figure 8D). There are two potential reasons for this. The first reason is that the correlation between TMB and the methylation level in different tumors is varied. The second reason is that the correlation between TMB and the methylation level in different subtypes is also well diversified. Further studies must be performed to verify this.

The correlation between mutation and DNA methylation is also important. A previous study reported that the hypomethylated blocks might promote mutation [90]. The methylation of cytosine can cause mutations to thymine [91]. However, another study reported that differential promoter methylation and somatic mutations interacted with each other in head and neck cancer [92]. The composition of the genes of mutations was different among the three subtypes. Figure 8. shows that there was less overlap among the three subtypes (Figure 8A–C). The mutations of ITGA9, ENG, EVI5, ATIC, and FZD2 in cluster 1 were significantly higher than those in the other subtypes. These genes are the biomarkers of mast cells, plasmacytoid dendritic cells, Type 2 T helper cells, immature dendritic cells, and macrophages [27].

The mutations of CTSZ, HOXA1, and KLRF1 in cluster 2 were significantly higher than those in the other subtypes. These genes are the biomarkers of natural killer cells, CD56 bright natural killer cells, and gamma delta T cells [27]. The mutations of DLC1, OSBPL1A, RRP12, C3AR1, MPZL1, and ITK in cluster 3 were significantly higher than those in the other subtypes. These genes are the biomarkers of Type 2 T helper cells, eosinophils, effector memory CD8 T cells, activated CD8 T cells, and activated CD4 T cells [27]. These immune cells with mutant genes were different among the three subgroups. The mutant genes could be promising drug targets.

Hypomethylated loci in cancer often coordinate with DNA break hotspots and may therefore contribute to copy number changes [90]. In Figure 7E, the CNV data in one subgroup was compared with the other two subgroups. AKNA with significant copy number gains was in cluster 1, and this gene is the biomarker of activated B cells. PARVG, SIK1, and UPK3A with significant copy number losses were in cluster 1, and these genes are the biomarkers of MDSC, effector memory CD8 T cells, and monocytes [27]. Figure 7F shows CLTB, GEMIN6, SIRPA, and SIRPG with significant copy number gains. These genes are the biomarkers of immature dendritic cells, activated CD8 T cells, plasmacytoid dendritic cells, and central memory CD4 T cells. DYRK2 with significant copy number losses was in cluster 2, and this gene is the biomarker of CD56 dim natural killer cells [27]. Likewise, CSF1R and GUSB with significant copy number gains were in cluster 3 (Figure 8G), and these genes are the biomarkers of T follicular helper cells and central memory CD8 T cells. CDC7, CHST12, CSF3R, and OGT with significant copy number losses were in cluster 3. These genes are the biomarkers of Type 2 T helper cells, T follicular helper cells, immature dendritic cells, and plasmacytoid dendritic cells [27]. These immune cells with mutant genes among the three subgroups were completely different. They also have a high chance as promising drug targets based on these CNV genes.

Triple-negative breast cancers with high tumor-infiltrating lymphocytes may show increased PD-L1 expression, which may be the reason that triple-negative breast cancers respond robustly to immune checkpoint inhibitor therapy [21]. Likewise, better responses to Atezolizumab were correlated with higher PD-L1 expression in the tumor-infiltrating leukocytes in BC [16]. In the current study, cluster 2 had the highest PD-L1 expression and cluster 1 had PD-L1 expression in the middle. This suggests that cluster 2 would be the most likely to respond to the PD-L1–Blocking Antibody and that cluster 1 might have a mid-range response to the PD-L1–Blocking Antibody.

Next, we analyzed chemotherapy’s impact on the three subgroups. Wang et al. reported that high immune scores were associated with therapeutic benefits from chemotherapy [23]. Similarly, breast cancer with high immune infiltration responded to chemotherapy, with pathologic complete response rates of 42% and 40% in the training cohort and validation cohort, respectively [56]. In contrast, those tumors without any immune infiltration had pathologically complete response rates of 3% and 7% in the training cohort and validation cohort, respectively [56]. A study reported that all subtypes of breast cancer with tumor-infiltrating lymphocytes benefited from chemotherapy, in particular, triple-negative breast cancers (TN) with >50% lymphocytic infiltration [21].

**Figure 9:**
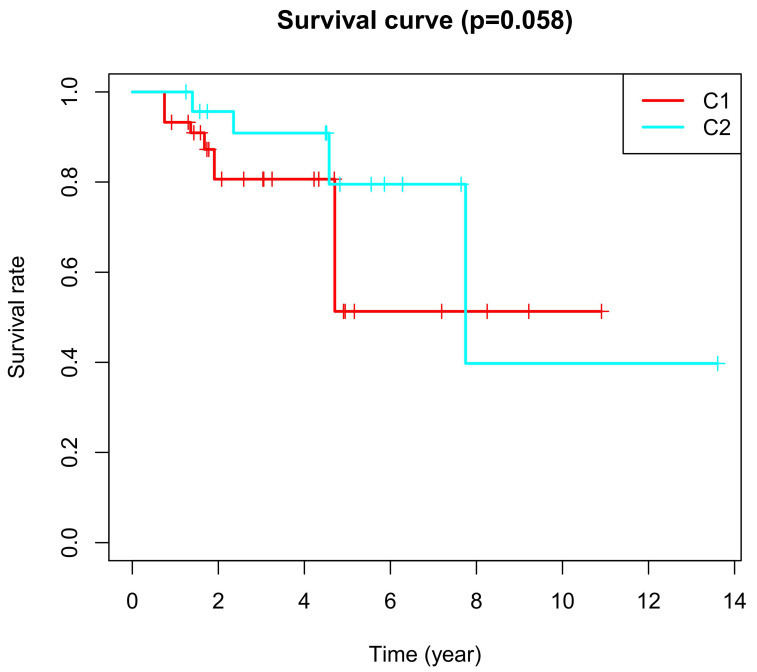
Kaplan–Meier curves. Kaplan–Meier curves based on CR and PR after chemotherapy with a log-rank test. C1 represents cluster 1 and C2 represents cluster 2.

The basal subtype of muscle-invasive bladder cancer with immune infiltration had a good response to chemotherapy [24,25]. One study reported the basal subtype with CD19+ tumor-infiltrating B-cells received a significant benefit from adjuvant chemotherapy [24]. Likewise, in the present study, cluster 2 had a greater response to chemotherapy compared with cluster 1, as shown in Table 1. Cluster 2 also had a better survival rate after chemotherapy, as shown in Figure 9 and Table 2.

All the above results suggest that cluster 2 is associated with more improvement in the overall survival after chemotherapy. There are two reasons: the first is that cluster 2 has high immune cell infiltration; the second is that cluster 2 has more basal markers (Figure 6). Then, we analyzed cluster 3. A previous study reported that the luminal subtype with low immune infiltration had a good survival with and without neoadjuvant chemotherapy [33]. In the present study, cluster 3 had the highest luminal marker expression and the lowest immune infiltration, and so this suggests that the cluster 3 might have a good survival rate with or without chemotherapy.

Finally, the three subgroups had specific methylation sites, specific DNA mutations, and CNV, as shown in Figure 8. All these will be promising targets for anti-cancer drug development. These results indicate that the anti-cancer targeted drug therapy are different among the three subgroups.

In conclusion, the classification was accurate and stable. BC patients were successfully divided into three subtypes based on the immune-cell-associated CpG sites. The three subgroups demonstrated different clinicopathologic features. The distribution of immune cells, level of expression of checkpoints, stromal score, immune score, ESTIMATEScore, tumor purity, APC co_inhibition, APC co_stimulation, HLA, MHC class I, Type I IFN response, and Type II IFN response demonstrated significant differences among the three subgroups. The distribution of genomic alterations was also different among the groups. High-level immune infiltration was correlated with high-level methylation. A lower RNAss was associated with higher immune infiltration and a higher level of expression of CD274. Cluster 2 was associated with a better response to chemotherapy. The anti-cancer targeted drug therapy are different among the three subgroups. The study of the intratumoral immune microenvironment may provide a new perspective for therapy in BC.

## 4. Materials and Methods

### 4.1. Data Pre-Processing

Four hundred and thirty-seven samples were used in this study. Methylation data using Illumina Human Methylation 450 arrays were obtained from UCSC Xena (https://xenabrowser.net/datapages/). DNAss, RNAss, and RNA-Seq from 430 BC samples and clinical data also were downloaded from the UCSC Xena website. The Masked Somatic Mutation data (MuTect2. Variant0. Maf) and the CNV data set (Masked Copy Number Segment, affymetrix snp 6.0) were collected from the TCGA website (https://portal.gdc.cancer.gov/repository). The CNV data comprised 814 samples. Due to the fact that the collected databases were public, the publishing policies of these databases were strictly obeyed by us, and ethical approval was not required.

### 4.2. Immune-Cell-Associated Gene Selection

Previous studies found that the DNA methylation sites in promoter regions strongly influenced gene expression [93,94]. The promoter regions were within 2 kb upstream to 0.5 kb downstream from the transcription start sites [93,94]. Immune-cell-associated biomarkers were obtained from previous studies (Table S1). Their corresponding methylation sites in promoter regions were obtained. The analysis showed that the exclusion probe criteria were as follows: (1) if the CpG site data had more than 70% of the samples missing, then the CpG sites were excluded from the analysis [95]. (2) Cross-reactive genome CpG sites were deleted [96]. (3) Probes on the X and Y chromosomes were excluded from the analysis [96]. The remaining sites were imputed with the k-nearest neighbors (KNN) imputation procedure [36].

### 4.3. Unsupervised Hierarchical Cluster Analysis

The methylation sites corresponding to immune-cell-associated genes were acquired. DMSs were identified between normal samples and bladder cancer samples with adjusted *p*-value < 0.05 and |deltabeta| > 0.2. Unsupervised hierarchical clustering was performed based on immune-cell-associated methylation data to identify subtypes of BC with the “sparcl” R software package (https://CRAN.R-project.org/package=sparcl). The overall survival curve of the BC subsets was obtained using the Kaplan–Meier method and with the “survival” package in R software. PCA was performed to validate the classification. A barplot was established to demonstrate the correlation between the clinical traits and the biological characteristics of the subtypes. Chi-square tests were performed, and *p* values less than 0.05 were considered significant.

### 4.4. Single Sample Gene Set Enrichment Analysis (ssGSEA) Based on Immune Cells Biomarker

ssGSEA was used to quantify the infiltration of immune cells that were obtained from the previous study [27]. The ssGSEA ranked the genes based on their absolute expression in a sample with the “GSEABase” and “GSVA” R packages. The enrichment score was calculated by integrating the differences between the empirical cumulative distribution functions of the gene ranks [97,98]. Activated B cells, activated CD8 T cells, effector memory CD8 T cells, central memory CD8 T cells, activated CD4 T cells, effector memory CD4 T cells, central memory CD4 T cells, regulatory T cells, gamma delta T cells, immature B cells, memory B cells, type 17 T helper cells, T follicular helper cells, type 1 T helper cells, and type 2 T helper cells are adaptive immune cells. CD56 dim natural killer cells, CD56 bright natural killer cells, eosinophils, activated dendritic cells, immature dendritic cells, MDSCs, macrophages, monocytes, mast cells, plasmacytoid dendritic cells, natural killer cells, natural killer T cells, and neutrophils are innate immune cells. Immune cells were compared among subgroups using the Kruskal–Wallis test. Apart from this, immune checkpoints were selected from previous studies [29,84] in order to compare them among the subtypes. Then, the Kruskal–Wallis test was performed.

### 4.5. Tumor Microenvironment (TME)

An algorithm called ESTIMATE was used for the estimation of stromal and immune cells in malignant tumor tissues based on the expression data [63]. The ESTIMATE algorithm was obtained from the public source website (https://sourceforge.net/projects/estimateproject/) to estimate the stromal scores and immune scores based on specific biomarkers associated with the infiltration of stromal and immune cells in tumor samples [62]. The stromal scores and immune scores were analyzed separately to predict the level of stromal and immune cells in tumor tissue, and these were combined to reference the tumor purity by ssGSEA [62,99,100]. The stromal scores, immune scores, tumor purity, and ESTIMATE scores were calculated for each sample. Subsequently, they were compared among the subtypes.

### 4.6. Single Sample Gene Set Enrichment Analysis (ssGSEA)

The biomarkers of APC_co_inhibition, APC_co_stimulation, endothelial cells, fibroblasts, HLA, inflammation-promoting, MHC_class_I, Type_I_IFN_Response, and Type_II_IFN_Response were selected from similar studies [82,101] (Table S2). Luminal biomarkers, basal biomarkers, squamous biomarkers, neuronal-differentiation biomarkers, and EMT-Claudin biomarkers were obtained from a previous study [30] (Table S3). Single sample gene set enrichment analysis was used to rank the genes based on their absolute expression in a sample.

### 4.7. DNAss and RNAss among Subgroups

The differentiated phenotype was rapidly lost during cancer progression, and progenitor and stem-cell-like characteristics were acquired [102]. The DNA hypermethylation of those genes suppressed gene expression, which was significantly benefited by cancer cells. Therefore, down-regulation of those genes may lead to the occurrence of cancer stem and progenitor cells by DNA hypermethylation [31,32]. RNAss based on mRNA expression and DNAss based on DNA methylation were utilized to measure the tumor stemness [88]. The range of scores was from 0 to 1. Zero indicates high differentiation, and 1 indicates undifferentiation [88]. Finally, the DNAss and RNAss among the three subgroups were analyzed.

### 4.8. Analysis of Mutations and CNVs among Subgroups

The ‘maftools’ software package was utilized to analyze and visualize the immune cell biomarker-associated mutation data [103]. The immune cell biomarker-associated mutation data were compared between one group and the other groups using the chi-square test. A *p*-value of less than 0.05 was considered significant. The TMB, which indicates the density of tumor gene mutations, was compared among subtypes based on the immune cell biomarker-associated mutation data.

The immune cell biomarker-associated CNV data were analyzed. The genomic identification of significant targets in cancer (GISTIC) algorithm was utilized to classify the copy number variant genes with remarkable gains and losses [104,105]. The parameter thresholds were adjusted to 0.2 and −0.2 for genomic gains and losses, respectively [104,105]. Immune cell biomarker-associated copy number variant data were compared between one group with the remaining groups using a chi-square test. A *p*-value of < 0.01 was considered significant.

## 5. Conclusions

The classification was accurate and stable. BC patients were successfully divided into three subtypes based on the immune-cell-associated CpG sites. Specific biological signaling pathways, immune mechanisms, and genomic alterations were found to have variations among the three subgroups. High-level immune infiltration was correlated with high-level methylation. The lower RNAss was associated with higher immune infiltration.

## Figures and Tables

**Table 1:** Response to chemotherapy. CPD represents clinical progressive disease, CR represents complete response, PR represents partial response, and SD represents stable disease.

Variant	Cluster 1 (Cases)	Cluster 2 (Cases)	Cluster 3 (Cases)
CPD	48	27	35
CR	55	46	16
PR	34	2	16
SD	30	2	9
☆ Unknown	7	19	65

☆ Unknown represents “unknown therapy or unknown the response to therapy”.

**Table 2:** Kaplan-Meier analyses. Kaplan–Meier analyses between cluster 1 and cluster 2.

Test	*p*-Value
Log Rank	0.058
Breslow	0.027
Tarone-ware	0.029

## Supplementary Materials

The following are available online at https://www.mdpi.com/2072-6694/12/10/3054/s1, Table S1: Immune cells-associated bio-markers. Table S2: Immune-related bio-markers. Table S3: The bio-markers of other classification.

## References

[1] Del Paggio J.C. (2018). Immunotherapy: Cancer immunotherapy and the value of cure. Nat. Rev. Clin. Oncol..

[2] Christofi T., Baritaki S., Falzone L., Libra M., Zaravinos A. (2019). Current perspectives in cancer immunotherapy. Cancers.

[3] Le D.T., Uram J.N., Wang H., Bartlett B.R., Kemberling H., Eyring A.D., Skora A.D., Luber B.S., Azad N.S., Laheru D. (2015). PD-1 blockade in tumors with mismatch-repair deficiency. N. Engl. J. Med..

[4] Reck M., Rodriguez-Abreu D., Robinson A.G., Hui R., Csöszi T., Fülöp A., Gottfried M., Peled N., Tafreshi A., Cuffe S. (2016). Pembrolizumab versus Chemotherapy for PD-L1-Positive Non-Small-Cell Lung Cancer. N. Engl. J. Med..

[5] Wolchok J.D., Kluger H., Callahan M.K., Postow M.A., Rizvi N.A., Lesokhin A.M., Segal N.H., Ariyan C.E., Gordon R.A., Reed K. (2013). Nivolumab plus Ipilimumab in advanced melanoma. N. Engl. J. Med..

[6] Mirjačić Martinović K.M., Vuletić A.M., Babović N.L., Džodić R.R., Konjević G.M., Jurišić V.B. (2017). Attenuated in vitro effects of IFN-α, IL-2 and IL-12 on functional and receptor characteristics of peripheral blood lymphocytes in metastatic melanoma patients. Cytokine.

[7] Chemin I. (2010). Evaluation of a hepatitis B vaccination program in Taiwan: Impact on hepatocellular carcinoma development. Future Oncol..

[8] Mammas I.N., Sourvinos G., Zaravinos A., Spandidos D.A. (2011). Vaccination against Human Papilloma Virus (HPV): Epidemiological Evidence of HPV in Non-genital Cancers. Pathol. Oncol. Res..

[9] Kantoff P.W., Higano C.S., Shore N.D., Berger E.R., Small E.J., Penson D.F., Redfern C.H., Ferrari A.C., Dreicer R., Sims R.B. (2010). Sipuleucel-T immunotherapy for castration-resistant prostate cancer. N. Engl. J. Med..

[10] Le D.T., Wang-Gillam A., Picozzi V., Greten T.F., Crocenzi T., Springett G., Morse M., Zeh H., Cohen D., Fine R.L. (2015). Safety and survival with GVAX pancreas prime and Listeria monocytogenes-expressing mesothelin (CRS-207) boost vaccines for metastatic pancreatic cancer. J. Clin. Oncol..

[11] Masson-Lecomte A., Rava M., Real F.X., Hartmann A., Allory Y., Malats N. (2014). Inflammatory biomarkers and bladder cancer prognosis: A systematic review. Eur. Urol..

[12] Kawai K., Miyazaki J., Joraku A., Nishiyama H., Akaza H. (2013). Bacillus Calmette-Guerin (BCG) immunotherapy for bladder cancer: Current understanding and perspectives on engineered BCG vaccine. Cancer Sci..

[13] Zhang C., Berndt-Paetz M., Neuhaus J. (2020). Identification of key biomarkers in bladder cancer: Evidence from a bioinformatics analysis. Diagnostics.

[14] Crispen P.L., Kusmartsev S. (2020). Mechanisms of immune evasion in bladder cancer. Cancer Immunol. Immunother..

[15] Babjuk M., Burger M., Compérat E.M., Gontero P., Mostafid A.H., Palou J., van Rhijn B.W.G., Rouprêt M., Shariat S.F., Sylvester R. (2019). European Association of Urology Guidelines on Non-muscle-invasive Bladder Cancer (TaT1 and Carcinoma in Situ)—2019 Update. Eur. Urol..

[16] Inman B.A., Longo T.A., Ramalingam S., Harrison M.R. (2017). Atezolizumab: A PD-L1-blocking antibody for bladder cancer. Clin. Cancer Res..

[17] Wołącewicz M., Hrynkiewicz R., Grywalska E., Suchojad T., Leksowski T., Roliński J., Niedźwiedzka-Rystwej P. (2020). Immunotherapy in bladder cancer: Current methods and future perspectives. Cancers.

[18] Jurkowska K., Długosz A. (2018). Research on new drugs in the therapy of bladder cancer (BC). Postepy Hig. Med. Dośw..

[19] Sharma P., Retz M., Siefker-Radtke A., Baron A., Necchi A., Bedke J., Plimack E.R., Vaena D., Grimm M.O., Bracarda S. (2017). Nivolumab in metastatic urothelial carcinoma after platinum therapy (CheckMate 275): A multicentre, single-arm, phase 2 trial. Lancet Oncol..

[20] Rosenberg J.E., Hoffman-Censits J., Powles T., Van Der Heijden M.S., Balar A.V., Necchi A., Dawson N., O’Donnell P.H., Balmanoukian A., Loriot Y. (2016). Atezolizumab in patients with locally advanced and metastatic urothelial carcinoma who have progressed following treatment with platinum-based chemotherapy: A single-arm, multicentre, phase 2 trial. Lancet.

[21] Stanton S.E., Disis M.L. (2016). Clinical significance of tumor-infiltrating lymphocytes in breast cancer. J. Immunother. Cancer.

[22] Asano Y., Kashiwagi S., Goto W., Takada K., Takahashi K., Hatano T., Takashima T., Tomita S., Motomura H., Ohsawa M. (2018). Prediction of treatment response to neoadjuvant chemotherapy in breast cancer by subtype using tumor-infiltrating lymphocytes. Anticancer Res..

[23] Wang S., Zhang Q., Yu C., Cao Y., Zuo Y., Yang L. (2020). Immune cell infiltration-based signature for prognosis and immunogenomic analysis in breast cancer. Brief. Bioinform..

[24] Jiang Q., Fu Q., Chang Y., Liu Z., Zhang J., Xu L., Zhu Y., Wang Y., Zhang W., Xu J. (2018). CD19 + tumor-infiltrating B-cells prime CD4 + T-cell immunity and predict platinum-based chemotherapy efficacy in muscle-invasive bladder cancer. Cancer Immunol. Immunother..

[25] Choi W., Porten S., Kim S., Willis D., Plimack E.R., Hoffman-Censits J., Roth B., Cheng T., Tran M., Lee I.L. (2014). Identification of Distinct Basal and Luminal Subtypes of Muscle-Invasive Bladder Cancer with Different Sensitivities to Frontline Chemotherapy. Cancer Cell.

[26] Wang Y., Ba H.J., Liu Z.C., Deng X.B., Zhou M. (2020). Prognostic value of immune cell infiltration in bladder cancer: A gene expression-based study. Oncol. Lett..

[27] Charoentong P., Finotello F., Angelova M., Mayer C., Efremova M., Rieder D., Hackl H., Trajanoski Z. (2017). Pan-cancer Immunogenomic Analyses Reveal Genotype-Immunophenotype Relationships and Predictors of Response to Checkpoint Blockade. Cell Rep..

[28] Zhang S., Wang Y., Gu Y., Zhu J., Ci C., Guo Z., Chen C., Wei Y., Lv W., Liu H. (2018). Specific breast cancer prognosis-subtype distinctions based on DNA methylation patterns. Mol. Oncol..

[29] Li W., Wang H., Ma Z., Zhang J., Ou-yang W., Qi Y., Liu J. (2019). Multi-omics Analysis of Microenvironment Characteristics and Immune Escape Mechanisms of Hepatocellular Carcinoma. Front. Oncol..

[30] Robertson A.G., Kim J., Al-Ahmadie H., Bellmunt J., Guo G., Cherniack A.D., Hinoue T., Laird P.W., Hoadley K.A., Akbani R. (2017). Comprehensive Molecular Characterization of Muscle-Invasive Bladder Cancer. Cell.

[31] Ohm J.E., McGarvey K.M., Yu X., Cheng L., Schuebel K.E., Cope L., Mohammad H.P., Chen W., Daniel V.C., Yu W. (2007). A stem cell-like chromatin pattern may predispose tumor suppressor genes to DNA hypermethylation and heritable silencing. Nat. Genet..

[32] Jones P.A., Baylin S.B. (2007). The Epigenomics of Cancer. Cell.

[33] Seiler R., Ashab H.A.D., Erho N., van Rhijn B.W.G., Winters B., Douglas J., Van Kessel K.E., Fransen van de Putte E.E., Sommerlad M., Wang N.Q. (2017). Impact of Molecular Subtypes in Muscle-invasive Bladder Cancer on Predicting Response and Survival after Neoadjuvant Chemotherapy [Figure presented]. Eur. Urol..

[34] Feinberg A.P. (2007). Phenotypic plasticity and the epigenetics of human disease. Nature.

[35] Laird P.W. (2003). The power and the promise of DNA methylation markers. Nat. Rev. Cancer.

[36] Yang C., Zhang Y., Xu X., Li W. (2019). Molecular subtypes based on DNA methylation predict prognosis in colon adenocarcinoma patients. Aging.

[37] Yang S., Wu Y., Wang S., Xu P., Deng Y., Wang M., Liu K., Tian T., Zhu Y., Li N. (2020). HPV-related methylation-based reclassification and risk stratification of cervical cancer. Mol. Oncol..

[38] Noushmehr H., Weisenberger D.J., Diefes K., Phillips H.S., Pujara K., Berman B.P., Pan F., Pelloski C.E., Sulman E.P., Bhat K.P. (2010). Identification of a CpG Island Methylator Phenotype that Defines a Distinct Subgroup of Glioma. Cancer Cell.

[39] Tian Z., Tian Z., Meng L., Meng L., Long X., Diao T., Hu M., Wang M., Liu M., Wang J. (2020). DNA methylation-based classification and identification of bladder cancer prognosis-associated subgroups. Cancer Cell Int..

[40] Yu Y., Cao H., Zhang M., Shi F., Wang R., Liu X. (2018). Prognostic value of DNA methylation for bladder cancer. Clin. Chim. Acta.

[41] Maruyama R., Toyooka S., Toyooka K.O., Harada K., Virmani A.K., Zöchbauer-Müller S., Farinas A.J., Minna J.D., Gazdar A.F., Virmani A.K. (2001). Aberrant promoter methylation profile of bladder cancer and its relationship to clinicopathological features. Cancer Res..

[42] Hao X., Luo H., Krawczyk M., Wei W., Wang W., Wang J., Flagg K., Hou J., Zhang H., Yi S. (2017). DNA methylation markers for diagnosis and prognosis of common cancers. Proc. Natl. Acad. Sci. USA.

[43] Arai E., Kanai Y. (2011). Genetic and epigenetic alterations during renal carcinogenesis. Int. J. Clin. Exp. Pathol..

[44] Chakraborty S., Panda A.K., Bose S., Roy D., Kajal K., Guha D., Sa G. (2017). Transcriptional regulation of FOXP3 requires integrated activation of both promoter and CNS regions in tumor-induced CD8+ Treg cells. Sci. Rep..

[45] Chen J., Guo X.Z., Li H.Y., Zhao J.J., Xu W. (2017). Da Dendritic cells engineered to secrete anti-DcR3 antibody augment cytotoxic T lymphocyte response against pancreatic cancer in vitro. World J. Gastroenterol..

[46] Medina P.J., Adams V.R. (2016). PD-1 Pathway Inhibitors: Immuno-Oncology Agents for Restoring Antitumor Immune Responses. Pharmacotherapy.

[47] Pan S., Zhan Y., Chen X., Wu B., Liu B. (2019). Bladder cancer exhibiting high immune infiltration shows the lowest response rate to immune checkpoint inhibitors. Front. Oncol..

[48] Hu B., Wang Z., Zeng H., Qi Y., Chen Y., Wang T., Wang J., Chang Y., Bai Q., Xia Y. (2020). Blockade of DC-SIGN+ Tumor-associated macrophages reactivates antitumor immunity and improves immunotherapy in muscle-invasive bladder cancer. Cancer Res..

[49] Podojil J.R., Glaser A.P., Baker D., Courtois E.T., Fantini D., Yu Y., Eaton V., Sivajothi S., Chiang M., Das A. (2020). Antibody targeting of B7-H4 enhances the immune response in urothelial carcinoma. Oncoimmunology.

[50] Fan M., Zhuang Q., Chen Y., Ding T., Yao H., Chen L., He X., Xu X. (2014). B7-H4 expression is correlated with tumor progression and clinical outcome in urothelial cell carcinoma. Int. J. Clin. Exp. Pathol..

[51] John P., Wei Y., Liu W., Du M., Guan F., Zang X. (2019). The B7x Immune Checkpoint Pathway: From Discovery to Clinical Trial. Trends Pharmacol. Sci..

[52] Chang C.S., Chang J.H., Hsu N.C., Lin H.Y., Chung C.Y. (2007). Expression of CD80 and CD86 costimulatory molecules are potential markers for better survival in nasopharyngeal carcinoma. BMC Cancer.

[53] McGranahan N., Rosenthal R., Hiley C.T., Rowan A.J., Watkins T.B.K., Wilson G.A., Birkbak N.J., Veeriah S., Van Loo P., Herrero J. (2017). Allele-Specific HLA Loss and Immune Escape in Lung Cancer Evolution. Cell.

[54] Bremnes R.M., Al-Shibli K., Donnem T., Sirera R., Al-Saad S., Andersen S., Stenvold H., Camps C., Busund L.T. (2011). The role of tumor-infiltrating immune cells and chronic inflammation at the tumor site on cancer development, progression, and prognosis: Emphasis on non-small cell lung cancer. J. Thorac. Oncol..

[55] Romero J.M., Jiménez P., Cabrera T., Cózar J.M., Pedrinaci S., Tallada M., Garrido F., Ruiz-Cabello F. (2005). Coordinated downregulation of the antigen presentation machinery and HLA class I/β2-microglobulin complex is responsible for HLA-ABC loss in bladder cancer. Int. J. Cancer.

[56] Denkert C., Loibl S., Noske A., Roller M., Müller B.M., Komor M., Budczies J., Darb-Esfahani S., Kronenwett R., Hanusch C. (2010). Tumor-associated lymphocytes as an independent predictor of response to neoadjuvant chemotherapy in breast cancer. J. Clin. Oncol..

[57] Melichar B., Študentová H., Kalábová H., Vitásková D., Čermáková P., Hornychová H., Ryška A. (2014). Predictive and prognostic significance of tumor-infiltrating lymphocytes in patients with breast cancer treated with neoadjuvant systemic therapy. Anticancer Res..

[58] Long M.D., Smiraglia D.J., Campbell M.J. (2017). The genomic impact of DNA CpG methylation on gene expression; relationships in prostate cancer. Biomolecules.

[59] Jeschke J., Bizet M., Desmedt C., Calonne E., Dedeurwaerder S., Garaud S., Koch A., Larsimont D., Salgado R., Van Den Eynden G. (2017). DNA methylation-based immune response signature improves patient diagnosis in multiple cancers. J. Clin. Investig..

[60] Bacolod M.D., Barany F., Fisher P.B. (2019). Can CpG methylation serve as surrogate markers for immune infiltration in cancer?. Adv. Cancer Res..

[61] Liang C., Yu X., Li B., Chen Y.A., Conejo-Garcia J., Wang X. (2019). DNA methylation-based immune cell deconvolution in solid tumors. bioRxiv.

[62] Hanahan D., Weinberg R.A. (2011). Hallmarks of cancer: The next generation. Cell.

[63] Yoshihara K., Shahmoradgoli M., Martínez E., Vegesna R., Kim H., Torres-Garcia W., Treviño V., Shen H., Laird P.W., Levine D.A. (2013). Inferring tumour purity and stromal and immune cell admixture from expression data. Nat. Commun..

[64] Chen W.Z., Jiang J.X., Yu X.Y., Xia W.J., Yu P.X., Wang K., Zhao Z.Y., Chen Z.G. (2019). Endothelial cells in colorectal cancer. World J. Gastrointest. Oncol..

[65] Tilki D., Kilic N., Sevinc S., Zywietz F., Stief C.G., Ergun S. (2007). Zone-specific remodeling of tumor blood vessels affects tumor growth. Cancer.

[66] Sherwood L.M., Parris E.E., Folkman J. (1971). Tumor Angiogenesis: Therapeutic Implications. N. Engl. J. Med..

[67] Ager A., Watson H.A., Wehenkel S.C., Mohammed R.N. (2016). Homing to solid cancers: A vascular checkpoint in adoptive cell therapy using CAR T-cells. Biochem. Soc. Trans..

[68] Bichsel C.A., Wang L., Froment L., Berezowska S., Müller S., Dorn P., Marti T.M., Peng R.W., Geiser T., Schmid R.A. (2017). Increased PD-L1 expression and IL-6 secretion characterize human lung tumor-derived perivascular-like cells that promote vascular leakage in a perfusable microvasculature model. Sci. Rep..

[69] Jennewein L., Bartsch G., Gust K., Kvasnicka H.M., Haferkamp A., Blaheta R., Mittelbronn M., Harter P.N., Mani J. (2018). Increased tumor vascularization is associated with the amount of immune competent PD-1 positive cells in testicular germ cell tumors. Oncol. Lett..

[70] Bagaria S.P., Gatalica Z., Maney T., Serie D., Parasramka M., Attia S., Krishna M., Joseph R.W. (2018). Association between programmed death-Ligand 1 expression and the vascular endothelial growth factor pathway in angiosarcoma. Front. Oncol..

[71] Motz G.T., Santoro S.P., Wang L.P., Garrabrant T., Lastra R.R., Hagemann I.S., Lal P., Feldman M.D., Benencia F., Coukos G. (2014). Tumor endothelium FasL establishes a selective immune barrier promoting tolerance in tumors. Nat. Med..

[72] Hendrix M.J.C., Seftor E.A., Hess A.R., Seftor R.E.B. (2003). Vasculogenic mimicry and tumour-cell plasticity: Lessons from melanoma. Nat. Rev. Cancer.

[73] Miyake M., Hori S., Morizawa Y., Tatsumi Y., Nakai Y., Anai S., Torimoto K., Aoki K., Tanaka N., Shimada K. (2016). CXCL1-Mediated Interaction of Cancer Cells with Tumor-Associated Macrophages and Cancer-Associated Fibroblasts Promotes Tumor Progression in Human Bladder Cancer. Neoplasia.

[74] Chow M.T., Möller A., Smyth M.J. (2012). Inflammation and immune surveillance in cancer. Semin. Cancer Biol..

[75] Ushijima T., Asada K. (2010). Aberrant DNA methylation in contrast with mutations. Cancer Sci..

[76] Hsieh C.J., Klump B., Holzmann K., Borchard F., Gregor M., Porschen R. (1998). Hypermethylation of the p16(INK4a) promoter in colectomy specimens of patients with long-standing and extensive ulcerative colitis. Cancer Res..

[77] Issa J.P.J., Ahuja N., Toyota M., Bronner M.P., Brentnall T.A. (2001). Accelerated age-related CpG island methylation in ulcerative colitis. Cancer Res..

[78] Eads C.A., Lord R.V., Kurumboor S.K., Wickramasinghe K., Skinner M.L., Long T.I., Peters J.H., DeMeester T.R., Danenberg K.D., Danenberg P.V. (2000). Fields of aberrant CpG island hypermethylation in Barrett’s esophagus and associated adenocarcinoma. Cancer Res..

[79] Kondo Y., Kanai Y., Sakamoto M., Mizokami M., Ueda R., Hirohashi S. (2000). Genetic instability and aberrant DNA methylation in chronic hepatitis and cirrhosis—A comprehensive study of loss of heterozygosity and microsatellite instability at 39 loci and DNA hypermethylation on 8 CpG islands in microdissected specimens from pati. Hepatology.

[80] Maekita T., Nakazawa K., Mihara M., Nakajima T., Yanaoka K., Iguchi M., Arii K., Kaneda A., Tsukamoto T., Tatematsu M. (2006). High levels of aberrant DNA methylation in Helicobacter pylori-infected gastric mucosae and its possible association with gastric cancer risk. Clin. Cancer Res..

[81] Nesi G., Nobili S., Cai T., Caini S., Santi R. (2015). Chronic inflammation in urothelial bladder cancer. Virchows Arch..

[82] Sui X., Lei L., Chen L., Xie T., Li X. (2017). Inflammatory microenvironment in the initiation and progression of bladder cancer. Oncotarget.

[83] He Y., Jiang Z., Chen C., Wang X. (2018). Classification of triple-negative breast cancers based on Immunogenomic profiling. J. Exp. Clin. Cancer Res..

[84] Zheng M., Hu Y., Gou R., Liu O., Nie X., Li X., Liu Q., Hao Y., Liu J., Lin B. (2020). Identification of immune-enhanced molecular subtype associated with BRCA1 mutations, immune checkpoints and clinical outcome in ovarian carcinoma. J. Cell. Mol. Med..

[85] de Vos L., Grünwald I., Bawden E.G., Dietrich J., Scheckenbach K., Wiek C., Zarbl R., Bootz F., Landsberg J., Dietrich D. (2020). The landscape of CD28, CD80, CD86, CTLA4, and ICOS DNA methylation in head and neck squamous cell carcinomas. Epigenetics.

[86] Lv D., Xing C., Cao L., Zhuo Y., Wu T., Gao N. (2020). PD-L1 gene promoter methylation represents a potential diagnostic marker in advanced gastric cancer. Oncol. Lett..

[87] Yim J., Koh J., Kim S., Song S.G., Ahn H.K., Kim Y.A., Jeon Y.K., Chung D.H. (2020). Effects of B7-H3 expression on tumour-infiltrating immune cells and clinicopathological characteristics in non–small-cell lung cancer. Eur. J. Cancer.

[88] Malta T.M., Sokolov A., Gentles A.J., Burzykowski T., Poisson L., Weinstein J.N., Kamińska B., Huelsken J., Omberg L., Gevaert O. (2018). Machine Learning Identifies Stemness Features Associated with Oncogenic Dedifferentiation. Cell.

[89] Cai L., Bai H., Duan J., Wang Z., Gao S., Wang D., Wang S., Jiang J., Han J., Tian Y. (2019). Epigenetic alterations are associated with tumor mutation burden in non-small cell lung cancer. J. Immunother. Cancer.

[90] Timp W., Feinberg A.P. (2013). Cancer as a dysregulated epigenome allowing cellular growth advantage at the expense of the host. Nat. Rev. Cancer.

[91] Bird A.P. (1996). The relationship of DNA methylation to cancer. Cancer Surv..

[92] Guerrero-Preston R., Michailidi C., Marchionni L., Pickering C.R., Frederick M.J., Myers J.N., Yegnasubramanian S., Hadar T., Noordhuis M.G., Zizkova V. (2014). Key tumor suppressor genes inactivated by “greater promoter” methylation and somatic mutations in head and neck cancer. Epigenetics.

[93] Chen X., Zhao C., Zhao Z., Wang H., Fang Z. (2019). Specific Glioma Prognostic Subtype Distinctions Based on DNA Methylation Patterns. Front. Genet..

[94] Jia D., Lin W., Tang H., Cheng Y., Xu K., He Y., Geng W., Dai Q. (2019). Integrative analysis of DNA methylation and gene expression to identify key epigenetic genes in glioblastoma. Aging.

[95] Li C., Ke J., Liu J., Su J. (2020). DNA methylation data–based molecular subtype classification related to the prognosis of patients with cervical cancer. J. Cell. Biochem..

[96] Chen Y.C., Elnitski L. (2019). Aberrant DNA methylation defines isoform usage in cancer, with functional implications. PLoS Comput. Biol..

[97] Finotello F., Trajanoski Z. (2018). Quantifying tumor-infiltrating immune cells from transcriptomics data. Cancer Immunol. Immunother..

[98] Bindea G., Mlecnik B., Tosolini M., Kirilovsky A., Waldner M., Obenauf A.C., Angell H., Fredriksen T., Lafontaine L., Berger A. (2013). Spatiotemporal dynamics of intratumoral immune cells reveal the immune landscape in human cancer. Immunity.

[99] Verhaak R.G.W., Hoadley K.A., Purdom E., Wang V., Qi Y., Wilkerson M.D., Miller C.R., Ding L., Golub T., Mesirov J.P. (2010). Integrated Genomic Analysis Identifies Clinically Relevant Subtypes of Glioblastoma Characterized by Abnormalities in PDGFRA, IDH1, EGFR, and NF1. Cancer Cell.

[100] Barbie D.A., Tamayo P., Boehm J.S., Kim S.Y., Moody S.E., Dunn I.F., Schinzel A.C., Sandy P., Meylan E., Scholl C. (2009). Systematic RNA interference reveals that oncogenic KRAS-driven cancers require TBK1. Nature.

[101] Xiao Y., Ma D., Zhao S., Suo C., Shi J., Xue M.Z., Ruan M., Wang H., Zhao J., Li Q. (2019). Multi-omics profiling reveals distinct microenvironment characterization and suggests immune escape mechanisms of triple-negative breast cancer. Clin. Cancer Res..

[102] Zhang X., Klamer B., Li J., Fernandez S., Li L. (2020). A pan-cancer study of class-3 semaphorins as therapeutic targets in cancer. BMC Med. Genom..

[103] Lv J., Zhu Y., Ji A., Zhang Q., Liao G. (2020). Mining TCGA database for tumor mutation burden and their clinical significance in bladder cancer. Biosci. Rep..

[104] Yang J., Chen Y., Luo H., Cai H. (2020). The Landscape of Somatic Copy Number Alterations in Head and Neck Squamous Cell Carcinoma. Front. Oncol..

[105] Luo H., Xu X., Yang J., Wang K., Wang C., Yang P., Cai H. (2020). Genome-wide somatic copy number alteration analysis and database construction for cervical cancer. Mol. Genet. Genom..

